# How does doxorubicin work?

**DOI:** 10.7554/eLife.00387

**Published:** 2012-12-18

**Authors:** Anand G Patel, Scott H Kaufmann

**Affiliations:** 1 **Anand G Patel** is in the Department of Molecular Pharmacology and Experimental Therapeutics, Mayo Clinic, Rochester, United States; 2 **Scott H Kaufmann** is in the Department of Molecular Pharmacology and Experimental Therapeutics and the Department of Oncology, Mayo Clinic, Rochester, United StatesKaufmann.Scott@mayo.edu

**Keywords:** doxorubicin, cancer, CREB3L1, ceramide, Human

## Abstract

A new mechanism involving cleavage of a transcription factor called CREB3L1 has been proposed to explain the anti-tumour effects of doxorubicin.

**Related research article** Denard B, Lee C, Ye J. 2012. Doxorubicin blocks proliferation of cancer cells through proteolytic activation of CREB3L1. *eLife* **1**:e00090. doi: 10.7554/eLife.00090

**Image** Cleavage of the transcription factor CREB3L1

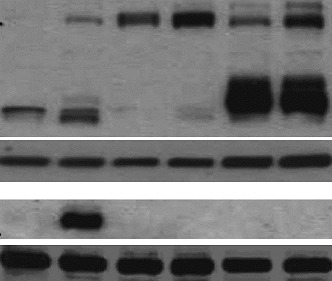

Many clinically available drugs are used without a clear understanding of how they work. This is particularly true in oncology, where powerful cytotoxic drugs are given to patients despite the fact that we do not fully understand their mechanism of action. Even targeted therapies such as rapamycins and receptor tyrosine kinase inhibitors are active in only a subset of the tumours for which they were originally developed, which has led to calls for better methods to identify tumours that will respond to specific drugs. This issue is even more pressing for conventional cytotoxic agents because, in addition to being toxic to cancer cells, they can also be toxic to healthy cells. Now, writing in *eLife*, Brey Denard, Ching Lee and Jin Ye provide evidence that doxorubicin, a widely used cancer drug, induces cellular toxicity via a novel mechanism that involves the synthesis of ceramide followed by activation of a transcription factor called CREB3L1 (Denard et al., 2012).

This is a story that began 40 years ago with the introduction of doxorubicin and daunorubicin into clinical practice. Both of these compounds belong to a class of drugs called anthracyclines that are derived from bacteria belonging to the genus *Streptomyces*. Extensive clinical studies have demonstrated that they are active against a wide variety of tumours (Minotti et al., 2004). However, despite this, the clinical use of anthracyclines has been limited because of a significant risk for cardiac damage. The chances of this life-threatening side effect depend on cumulative dosage, and can occur decades after exposure (Kremer et al., 2001). Over the years, many mechanisms of action have been proposed for these drugs—including topoisomerase II inhibition, DNA intercalation, and free radical generation—but there has until recently been a lack of definitive evidence for all three of these mechanisms (Gewirtz, 1999).

Denard, Lee and Ye, who are based at the University of Texas Southwestern (UTSW) Medical Center, propose that doxorubicin can combat tumours via a mechanism called regulated intramembrane proteolysis. In this process, a membrane-bound protein is cleaved, liberating a soluble messaging molecule that can play a role in a variety of cellular processes, including apoptosis, lipid metabolism and the response to viral infection (Lal and Caplan, 2011).

In previous work, the UTSW group showed that CREB3L1—a membrane protein with a carboxy-domain inside the lumen of the endoplasmic reticulum, and an amino-terminal domain that is in the cytoplasm of the cell—undergoes proteolytic cleavage during viral infection to release the amino-terminal domain, which then translocates to the nucleus and drives the transcription of genes that inhibit cellular proliferation (Denard et al., 2011). In the present study, Denard, Lee and Ye extend these results by treating multiple cell lines with doxorubicin and showing that the drug produces lipid molecules called ceramides that trigger the cleavage of CREB3L1 (see Figure 1). This is again followed by translocation of the resulting amino-terminal fragment to the nucleus and the increased expression of several genes (Denard et al., 2012). Importantly, the down-regulation of CREB3L1 diminishes doxorubicin-induced effects on cell proliferation, whereas overexpression of CREB3L1 makes tumour cells more sensitive to doxorubicin. Collectively, these observations provide the first evidence for a model in which the anthracycline-induced synthesis of ceramides leads to the cleavage of CREB3L1, resulting in altered expression of genes that might contribute to the effect of the drug.

**Figure 1.**
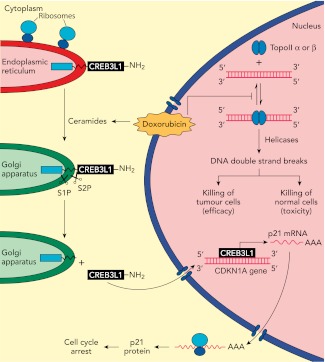
Understanding how the anticancer drug doxorubicin works is an important challenge in cancer research. Two independent groups have recently published evidence for two potential mechanisms that might be able to explain anti-tumour effects of doxorubicin. Zhang et al. showed diminished toxicity in cardiomyocytes from mice lacking the *Top2b* gene. These data are consistent with previous claims that doxorubicin helps to stabilize complexes containing double-stranded DNA and the enzyme topoisomerase II (top right of figure): this enzyme then cuts both of the DNA strands, which leads to the death of both normal cells (predominantly via topoisomerase IIβ) and in tumour cells that are susceptible to the drug (predominantly via topoisomerase IIα), thus accounting for both the toxicity and anti-tumour efficacy of doxorubicin. Denard et al. propose that doxorubicin increases the production of ceramides inside cells (top left), which leads to the latent transcription factor CREB3L1 translocating from the endoplasmic reticulum to the Golgi apparatus. Two proteases (S1P and S2P) then cut the CREB3L1 protein; and its amino-terminal fragment then migrates into the nucleus, where it acts as a transcription factor to activate the CDNK1A locus and additional genes (bottom right). This leads to increased expression of the p21 protein along with other proteins that inhibit the proliferation of tumour cells.

While intriguing, these results represent only a first step in testing this model. In particular, as discussed by the authors, there are several open questions: Does ceramide alter CREB3L1 trafficking to the Golgi apparatus, where the intramembrane proteases are located? Does *CDKN1A*, one of the genes that is overexpressed when cells are treated with doxorubicin, have a critical role in the action of the drug? And does expression or activation of CREB3L1 correlate with doxorubicin sensitivity across a panel of cancer cell lines, such as the Cancer Cell Line Encyclopedia (Barretina et al., 2012), or clinical specimens? In addition, it will be important to establish whether other chemo-therapeutic agents also induce proteolysis of CREB3L1 or other transmembrane proteins.

It will also be interesting to see whether the ideas put forward by the UTSW group can be integrated into existing models of doxorubicin action. Emerging data suggest that the enzyme topoisomerase IIβ has a critical role in the toxic effects of doxorubicin on cardiac tissue (see Figure 1), and it has been reported that the targeted deletion of the *Top2b* gene abolishes doxorubicin-induced effects on cardiac function (Zhang et al., 2012). By contrast, it has been suggested that the *Top2a* gene contributes to the anticancer effects of doxorubicin and other anthracyclines (Mao et al., 1999). This work, and also the work of the UTSW group, needs to be rigorously extended by studying in detail how doxorubicin kills cancer cells in appropriate cancer models and clinical samples.

The fact that doxorubicin remains the subject of intense study four decades after its introduction into the clinic is a reflection of its complex biology and its importance as a cancer drug. Hopefully, these recent studies will ultimately allow clinicians to better understand this important agent and to utilize it in a more targeted fashion.

## References

[bib1] Barretina J Caponigro G Stransky N Venkatesan K Margolin AA Kim S 2012 The Cancer Cell Line Encyclopedia enables predictive modelling of anticancer drug sensitivity. Nature 483:603–7 doi: 10.1038/nature11003 22460905 PMC3320027

[bib2] Denard B Seemann J Chen Q Gay A Huang H Chen Y 2011 The membrane-bound transcription factor CREB3L1 is activated in response to virus infection to inhibit proliferation of virus-infected cells. Cell Host Microbe 10:65–74 doi: 10.1016/j.chom.2011.06.006 21767813 PMC3139916

[bib3] Denard B Lee C Ye J 2012 Doxorubicin blocks proliferation of cancer cells through proteolytic activation of CREB3L1. eLife 1:e00090 doi: 10.7554/elife.00090 PMC3524649 23256041

[bib4] Gewirtz D 1999 A critical evaluation of the mechanisms of action proposed for the antitumor effects of the anthracycline antibiotics adriamycin and daunorubicin. Biochem Pharmacol. 57:727–41 doi: 10.1016/S0006-2952(98)00307-4 10075079

[bib5] Kremer LC van Dalen EC Offringa M Ottenkamp J Voute PA 2001 Anthracycline-induced clinical heart failure in a cohort of 607 children: long-term follow-up study. J Clin Oncol 19:191–6 11134212 10.1200/JCO.2001.19.1.191

[bib6] Lal M Caplan M 2011 Regulated intramembrane proteolysis: signaling pathways and biological functions. Physiology (Bethesda) 26:34–44 doi: 10.1152/physiol.00028.2010 21357901

[bib7] Mao Y Yu C Hsieh TS Nitiss JL Liu AA Wang H 1999 Mutations of human topoisomerase II alpha affecting multidrug resistance and sensitivity. Biochemistry 38:10793–800 doi: 10.1021/bi9909804 10451375

[bib8] Minotti G Menna P Salvatorelli E Cairo G Gianni L 2004 Anthracyclines: molecular advances and pharmacologic developments in antitumor activity and cardiotoxicity. Pharmacol Rev 56:185–229 doi: 10.1124/pr.56.2.6 15169927

[bib9] Zhang S Liu X Bawa-Khalfe T Lu LS Lyu YL Liu LF 2012 Identification of the molecular basis of doxorubicin-induced cardiotoxicity. Nat Med. 18:1639–42 doi: 10.1038/nm.2919 23104132

